# Assessment of the Dissemination of COVID-19–Related Articles Across Social Media: Altmetrics Study

**DOI:** 10.2196/41388

**Published:** 2023-07-12

**Authors:** Haley Tornberg, Carine Moezinia, Chapman Wei, Simone A Bernstein, Chaplin Wei, Refka Al-Beyati, Theodore Quan, David J Diemert

**Affiliations:** 1 Cooper Medical School of Rowan University Camden, NJ United States; 2 Department of Medicine, Hospital for Special Surgery New York, NY United States; 3 Department of Medicine, Staten Island University Hospital Staten Island, NY United States; 4 Department of Psychiatry Barnes-Jewish Hospital Washington University in St. Louis School of Medicine St. Louis, MO United States; 5 American University of Antigua Coolidge Antigua and Barbuda; 6 Department of Medicine David Geffen School of Medicine at University of California Los Angeles, CA United States; 7 The George Washington School of Medicine and Health Sciences Washington, DC United States

**Keywords:** Altmetric, COVID-19, citation, dissemination, information spread, impact factor, information, social media, bibliometric, scientometric, health professional, Twitter, database, data, citation, impact factor

## Abstract

**Background:**

The use of social media assists in the distribution of information about COVID-19 to the general public and health professionals. Alternative-level metrics (ie, Altmetrics) is an alternative method to traditional bibliometrics that assess the extent of dissemination of a scientific article on social media platforms.

**Objective:**

Our study objective was to characterize and compare traditional bibliometrics (citation count) with newer metrics (Altmetric Attention Score [AAS]) of the top 100 Altmetric-scored articles on COVID-19.

**Methods:**

The top 100 articles with the highest AAS were identified using the Altmetric explorer in May 2020. AAS, journal name, and mentions from various social media platforms (Twitter, Facebook, Wikipedia, Reddit, Mendeley, and Dimension) were collected for each article. Citation counts were collected from the Scopus database.

**Results:**

The median AAS and citation count were 4922.50 and 24.00, respectively. *The* *New England Journal of Medicine* published the most articles (18/100, 18%). Twitter was the most frequently used social media platform with 985,429 of 1,022,975 (96.3%) mentions. Positive correlations were observed between AAS and citation count (*r*^2^=0.0973; *P*=.002).

**Conclusions:**

Our research characterized the top 100 COVID-19–related articles by AAS in the Altmetric database. Altmetrics could complement traditional citation count when assessing the dissemination of an article regarding COVID-19.

**International Registered Report Identifier (IRRID):**

RR2-10.2196/21408

## Introduction

SARS-CoV-2 is the pathogen responsible for the latest global pandemic that has exhausted the global economy and health care system to a degree that has not been seen since the 1918 influenza outbreak. Originating from Wuhan, China, in December 2019, more than 230,000 reported fatalities have occurred worldwide as of May 1, 2020, from COVID-19, which is more than that from both severe acute respiratory syndrome and Middle East respiratory syndrome combined [1,2]. Due to its rapid spread and massive casualties, there has been a rapid rate of research dissemination across medical journals, and social media platforms to provide real-time guidance to the understanding of the epidemiology, disease characteristics, clinical management, and future treatment developments for COVID-19 to all stakeholders invested in managing this pandemic [3,4].

In contrast to medical journals, social media can serve as a useful platform to inform the wider general public, both medical professionals and laypeople alike, and to disseminate crucial and novel information during this evolving crisis [5,6]. To capture the level of an article’s dissemination across social media, or the “online attention” it receives, alternative metric tools, such as Altmetric, have been created [7-9]. As opposed to traditional metrics of article dissemination, such as article citation count, metrics that capture article dissemination across social media are known as alternative metrics, or “altmetrics.” Provided by Altmetric, one of the main platforms of altmetrics that comprehensively measures web-based social media activity associated with academic articles, the Altmetric Attention Score (AAS) is a weighted score of the amount of “online attention” a research article has received across social media platforms. This refers solely to the number of citations, link-outs, and abstract views. These platforms include Twitter, Facebook, Google+, Wikipedia, blogs, and many others [10].

Given that AAS uses relevant social media platforms, including Twitter and Facebook, Altmetric can be a potentially useful adjunctive bibliometric tool for holistically evaluating an article’s “impact” beyond just scholarly impact [11]. This includes information uptake, information engagement, and relevance of results. While the citation count or impact factor reflects the number of citations in other articles or journals, altmetrics reflect the instantaneous attention an article garners among news outlets, blogs, Twitter, Facebook, and other media platforms [4]. Additionally, as paper journals move toward web-based platforms, the armament of these new internet-based technologies provides researchers with a new approach to assessing the effect of research [4]. Over the years in biomedical research, the use of Twitter to disseminate article information has increased drastically, showcasing the degree to which the medical community uses social media [12]. Previous studies have assessed the utility of these tools in determining its complementary use with citation count in various medical fields, but have reported variable results [4,11,13,14].

However, the utility of altmetrics in relation to COVID-19 research has not yet been evaluated [15]. Given that there has been a massive influx of COVID-19–related publications since early 2020, and that altmetrics allow one to rapidly assess an article’s level of dissemination upon publication, altmetrics have the potential to be used complementarily with traditional bibliometrics such as article citation for assessing COVID-19–related research [10]. In the age of the internet where both the academic and the general communities are searching for insightful articles about COVID-19 on the web to gain a better understanding, altmetrics can provide a broader view of an article’s web-based interest and scholarly impact [16]. The purpose of this study was to identify and characterize the 100 most “trended” COVID-19–related articles (ie, those with the highest AAS scores) across social media captured by Altmetric.

## Methods

Altmetric Explorer was used to identify COVID-19–related publications from December 2019 to May 2020 using the PubMed search terms “COVID-19,” “SARS CoV-2,” and “coronavirus.” Articles that were retracted by May 8, 2020, were excluded from the analysis. In total, 926 articles were found at that time. From this list, the top 100 articles were selected on the basis of their AAS and analyzed on May 8, 2020. The number of mentions from the following Altmetric data components were extracted and examined: news mentions, blog mentions, policy mentions, Twitter mentions, Facebook mentions, Wikipedia mentions, Reddit mentions, Mendeley readers, and the number of Dimension citations [17]. Both Mendeley and Dimensions are research platforms that allow one to share research output. Altmetric can retrieve the number of Mendeley members, or “Mendeley readers,” who read a particular article [18]. Similarly, Altmetric can extract the total references between publications from existing databases and full-text records available on the Dimensions database, or “Dimension citation” [19]. In addition to the extraction of Altmetric data components, we also collected data about where the articles were published, article type, country of article origin, and the article citation count [10,12]. The country of origin was determined from the author affiliation. If authors were from more than 1 country, the article was considered as describing an “international cooperation.” For traditional citation analysis, the article citation count was determined using the Scopus database.

## Results

Table 1 summarizes the altmetrics of the top 100 Altmetric articles. A majority of articles were published in biomedical journals. Of these 100 articles, The New England Journal of Medicine published the most manuscripts (18/100, 18%). In total, 42 of 100 (42%) articles were original investigations. Articles from The New England Journal of Medicine had the most news mentions (n=7073), blog mentions (n=637), and blog policy mentions (n=52) regarding COVID-19 (Multimedia Appendix 1). The article with the highest AAS (33,828) was a biomechanistic basic science letter delineating the features of the SARS-CoV-2 genome and providing evidence that SARS-CoV-2 was not constructed in a laboratory (AAS=33,828; citation count=30), while the article with the highest citation count was one of the largest case series that assessed the characteristics of patients admitted to the hospital for COVID-19 pneumonia in China (AAS=14,276; citation count=1,096; Multimedia Appendix 2) [20]. Median AAS, Field-Weighted Citation Impact, and citation count were 4,922.50, 37.92, and 24.00, respectively. All articles were classified in the top 5% of scientific output [12], implying that these articles rank in the top 5% of more than 15.5 million research publications scored by Altmetrics. In other words, these articles have garnered the most attention. A majority of articles originated from China (n=32), followed by the United States (n=27; Table 2). In total, 22 articles were published by authors affiliated with institutions that have international cooperation. The article with the most Mendeley readers was a viewpoint article summarizing one of the largest case series describing patient characteristics from China (Mendeley reader=2581) [21]. The article with the most Dimension citations was the same study that had the highest citation count (Dimension citation number=2233) [22]. There was a total of 1,022,975 mentions of the social media platforms that were assessed; of these, Twitter had the most mentions of the selected articles at 96.3% (985,429/1,022,975). Additionally, 99 articles were open access. A positive correlation was observed between AAS and citation count (r2=0.0973; *P*=.002).

**Table 1:** Characteristics and components of the Top 100 COVID-19–related articles by Altmetric score^a^.

Characteristics		Values^b^
Altmetric score, median (range)		4922.50 (2841-33,828)
Traditional citation, median (range)^c^		24.00 (0-1096)
News mentions, n (range)		32,509 (1-2021)
Blog mentions, n (range)		2630 (0-131)
Policy mentions, n (range)		154 (0-21)
Twitter mentions, n (range)		985,429 (1381-84,022)
Facebook mentions, n (range)		1138 (0-58)
Wikipedia mentions, n (range)		177 (0-11)
Reddit mentions, n (range)		938 (0-40)
Mendeley readers, n (range)		9283 (0-2581)
Dimension citations, n (range)		18,011 (0-2233)
**Article types, n (%)**		
	Original investigation	44 (44)
	Correspondence	31 (31)
	Editorial	14 (14)
	Review	4 (4)
	Viewpoint	6 (6)
	Open access	100 (100)
**Study design of original investigations (n=44), n (%)**		
	Clinical trials	4 (9)
	Prospective or retrospective cohort studies	21 (48)
	Cross-sectional	1 (2)
	Case series	7 (16)
	Basic science in vitro or in vivo studies	8 (18)
	Model validation studies	3 (7)

^a^A total of 16 articles had a Field-Weighted Citation Impact of 0 because they did not have a score yet on Scopus at the time of the study and were not part of the analysis.

^b^Total sum of all mentions for each article included in the study.

^c^Three articles did not have a citation count on Scopus or PubMed Central at the time of the study and were not part of the analysis.

**Table 2:** Top 100 Articles with the highest Altmetric Attention Scores according to country of origin.

Country	Articles, n
United States	27
China	32
United Kingdom	6
Germany	4
Italy	3
France	2
Australia	1
Iceland	1
Singapore	1
South Korea	1
International collaboration	22

## Discussion

In the past several months since COVID-19 has spread globally, many articles pertaining to the pandemic have been disseminated. Traditionally, high-quality and regarded articles can be ascertained via citation count; however, that is not always possible in a rapidly evolving pandemic such as the current one. This study identified and characterized the top 100 Altmetric articles related to COVID-19.

A majority of these top studies disseminated across social media platforms originated from authors from China. This is not surprising as COVID-19 originated from Wuhan, China. Within the 5-6 months that COVID-19 was discovered, the general public, as well as academic readers, was engaged in learning about the clinical characteristics of COVID-19 in China. It was surprising that the article that received the most attention per Altmetric was a correspondence letter analyzing the key features of the SARS-CoV-2 genome and theories of its origin [20]. This suggests that the public’s engagement in COVID-19–related research was increased in understanding the origin of the COVID-19 pandemic at that time. From a bibliometric perspective, *The New England Journal of Medicine* has been publishing articles with the highest collective AAS. This is not surprising as *The New England Journal of Medicine* is the highest-ranked general medicine journal [23]. Hence, the general public disseminates articles that are published in highly reputed journals across social media platforms the most.

Studies have previously shown that Altmetric scores are positively correlated with traditional citation count [14,24-28]. In the current literature, citation count has shown a weakly positive correlation with AAS. Interestingly, the current literature notes that journals with a high Twitter presence have a higher AAS, which our data also reinforce [29]. In our analysis, we observed that Twitter was the most frequently used social media platform and had the most mentions of our selected articles, which also aligns with previous findings [30]. All these studies have stated that altmetrics, including AAS, are most effectively used complementary to traditional bibliometrics and should not necessarily be used on their own to assess an article’s quality [4,7,10,11].

Interestingly, it is important to note that original articles, particularly observational cohort studies, were disseminated more quickly on social media platforms, while the top biomechanistic basic science article was also rapidly disseminated, most likely because this article addressed the possibility of SARS-CoV-2 being engineered in a laboratory [20]. Additionally, all the articles were open access and freely accessible to the general public likely because there is an alignment between scientists and the general public concerning the need to disseminate new findings related to COVID-19 as quickly as possible, given the enormous burden of this ongoing pandemic. With the access barrier removed, there is significantly greater availability than usual to read newly published articles and then disseminate them to peers.

There are several limitations to our study. This study analyzed the trend of COVID-19–related studies at the beginning of the COVID-19 pandemic to show and assess what articles were extremely relevant to the general viewer at that time. Article types can change over time, especially when the COVID-19 pandemic is over. Additionally, our study only assessed the top 100 articles, which could have resulted in a selection bias. Other articles were not included for analysis because were not as trendy. Thus, the results may change with additional studies. We also only used Altmetric for analysis. Other altmetrics tools, including PlumX and Impact story, may use different algorithms in determining web-based impact. Altmetrics do not necessarily reflect the scientific quality of an article [14,31]. Just because an article generated more attention among the general public and academic community, it does not correlate with a better study design, results, or quality of evidence. AAS is more dynamic relative to citation count, making the precision, consistency, and reproducibility of Altmetric analysis challenging.

Our study did not evaluate altmetrics as part of a bigger image with epistemological and sociological tools outside of the platform information provided in Altmetrics Explorer. Future research in that direction may help enhance our understanding of altmetrics in COVID-19–related research.

In conclusion, our study characterized an early general public engagement of the top 100 Altmetric articles related to COVID-19. The top-most published articles shared across various platforms focused on the clinical characteristics of COVID-19 and exploring the origin of SARS-CoV-2. While altmetrics and citation count were weakly correlated, these 2 metrics are separate and unique such that they may augment the holistic understanding of an article’s impact when juxtaposed with each other.

## Appendix

Multimedia Appendix 1 Comparison of journals and their total scores and mentions.

Multimedia Appendix 2 Articles and their Altmetric score and mentions.

## Abbreviations

AAS: Altmetric Attention Score
