# Supplementary material for: Assessment of the Dissemination of COVID-19–Related Articles Across Social Media: Altmetrics Study
Source: JMIR Form Res. 2023 Jul 12;7:e41388. doi: 10.2196/41388 (PMC10365589; doi:10.2196/41388)
Supplement: Multimedia Appendix 1 [file formative_v7i1e41388_app1.docx]

| *Journal* | *# of Papers* | *Altmetric Attention Score* | *Citation Count* | *Article Types* | *# of News Mentions* | *# of Blog Mentions* | *# of Policy Mentions* | *# of Twitter Mentions* | *# of Facebook Mentions* | *# of Wikipedia Mentions* | *# of Reddit Mentions* | *# of Mendeley Citations* | *# of Dimension Citations* |
| --- | --- | --- | --- | --- | --- | --- | --- | --- | --- | --- | --- | --- | --- |
| ACS Nano | 1 | 2958 | 0 | 1 Original | 156 | 13 | 0 | 3,553 | 1 | 0 | 5 | 0 | 0 |
| Annals of Internal Medicine | 2 | 11,385 | 65 | 1 Original, 1 Editorial | 1,265 | 69 | 2 | 11,135 | 13 | 0 | 0 | 0 | 194 |
| Antiviral Research | 1 | 12244 | 7 | 1 Original | 300 | 14 | 0 | 18,697 | 9 | 3 | 29 | 0 | 23 |
| BioScience Trends | 1 | 4,335 | 112 | 1 Editorial | 155 | 16 | 1 | 5,385 | 17 | 1 | 3 | 0 | 210 |
| British Medical Journal | 2 | 9,551 | 16 | 2 Editorial | 170 | 25 | 1 | 13,731 | 27 | 1 | 20 | 0 | 32 |
| Cell | 1 | 3,964 | 113 | 1 Original | 156 | 27 | 0 | 5,813 | 15 | 3 | 33 | 0 | 377 |
| Cell Discovery | 1 | 3,244 | 32 | 1 Correspondence | 100 | 2 | 0 | 3,664 | 4 | 0 | 16 | 0 | 0 |
| Cell Research | 1 | 6,979 | 201 | 1 Correspondence | 393 | 59 | 1 | 9,421 | 23 | 8 | 14 | 0 | 425 |
| Circulation: Arrhythmia and Electrophysiology | 1 | 4,229 | 0 | 1 Original | 1 | 1 | 0 | 22,468 | 0 | 0 | 5 | 0 | 0 |
| EBioMedicine | 1 | 4,018 | 1 | 1 Original | 372 | 19 | 0 | 2,506 | 2 | 1 | 4 | 0 | 7 |
| Emerging Infectious Diseases | 4 | 31,438 | 8 | 1 Correspondence, 3 Original | 720 | 30 | 0 | 57,029 | 11 | 4 | 56 | 0 | 30 |
| Engineering | 1 | 3,777 | 6 | 1 Original | 11 | 0 | 0 | 10,115 | 0 | 1 | 2 | 0 | 14 |
| Eurosurveillance | 1 | 3,638 | 26 | 1 Original | 476 | 27 | 1 | 1,381 | 2 | 0 | 16 | 0 | 102 |
| International Journal of Antimicrobial Agents | 1 | 7,869 | 60 | 1 Original | 417 | 58 | 0 | 9,222 | 9 | 3 | 3 | 0 | 244 |
| JAMA Network Open | 1 | 2,870 | 28 | 1 Original | 200 | 23 | 0 | 2,204 | 4 | 0 | 3 | 754 | 64 |
| JAMA: Journal of the American Medical Association | 12 | 75,310 | 1,343 | 4 Original, 4 Viewpoint, 1 Editorial, 3 Correspondence | 3,674 | 293 | 9 | 134,601 | 143 | 11 | 98 | 8,300 | 1,737 |
| Journal of Hospital Infection | 1 | 12,520 | 80 | 1 Systematic Review | 743 | 28 | 8 | 14,401 | 40 | 4 | 40 | 0 | 140 |
| Journal of Medical Virology | 2 | 8,996 | 109 | 1 Review, 1 Original | 574 | 42 | 1 | 10,950 | 26 | 0 | 38 | 0 | 209 |
| Journal of Travel Medicine | 1 | 5,684 | 11 | 1 Original | 29 | 7 | 0 | 29,161 | 2 | 1 | 4 | 0 | 28 |
| Lancet Infectious Diseases | 3 | 13,928 | 33 | 2 Original, 1 Correspondence | 612 | 53 | 3 | 20,702 | 5 | 1 | 15 | 0 | 119 |
| Medecine & Maladies Infectieuses | 1 | 5,557 | 12 | 1 Correspondence | 120 | 22 | 0 | 8,674 | 2 | 0 | 13 | 0 | 29 |
| MMWR: Morbidity & Mortality Weekly Report | 8 | 38,487 | 56 | 8 Correspondence | 3,699 | 207 | 17 | 36,610 | 64 | 3 | 38 | 1 | 200 |
| Nature | 2 | 10,538 | 302 | 1 Original, 1 Correspondence | 816 | 67 | 0 | 13,000 | 22 | 12 | 13 | 0 | 959 |
| Nature Biotechnology | 1 | 2,876 | 0 | 1 Original | 114 | 10 | 0 | 3,846 | 7 | 0 | 13 | 0 | 2 |
| Nature Medicine | 5 | 53,893 | 56 | 1 Original, 4 Correspondence | 1,798 | 163 | 0 | 116,895 | 61 | 11 | 52 | 0 | 212 |
| New England Journal of Medicine | 18 | 138,466 | 3,127 | 5 Correspondence, 4 Editorial, 8 Original, 1 Viewpoint | 7,073 | 637 | 52 | 197,835 | 333 | 47 | 170 | 165 | 5,739 |
| Pediatrics | 1 | 6,667 | 52 | 1 Original | 566 | 35 | 1 | 6,429 | 6 | 0 | 5 | 0 | 113 |
| Proceedings of the National Academy of Sciences of the United States of America | 2 | 11,711 | 8 | 1 Original, 1 Correspondence | 520 | 26 | 0 | 16,769 | 4 | 3 | 18 | 0 | 34 |
| Science | 6 | 38,933 | 166 | 1 Correspondence, 1 Report, 1 Editorial, 3 Original | 1,407 | 164 | 4 | 58,420 | 59 | 12 | 63 | 7 | 313 |
| The Lancet | 14 | 85,635 | 3,321 | 2 Correspondence, 3 Editorial, 7 Original, 1 Review, 1 Viewpoint | 5,126 | 430 | 53 | 123,398 | 204 | 44 | 117 | 56 | 6,272 |
| The Lancet Child & Adolescent Health | 1 | 4,426 | 0 | 1 Review | 238 | 10 | 0 | 4,923 | 1 | 0 | 1 | 0 | 3 |
| The Lancet Respiratory Medicine | 2 | 10,666 | 82 | 1 Correspondence, 1 Editorial | 508 | 53 | 0 | 12,491 | 22 | 3 | 31 | 0 | 180 |

Table S1. Comparison of Journals and Their Total Scores and Mentions
