# Supplementary material for: Assessment of the Dissemination of COVID-19–Related Articles Across Social Media: Altmetrics Study
Source: JMIR Form Res. 2023 Jul 12;7:e41388. doi: 10.2196/41388 (PMC10365589; doi:10.2196/41388)
Supplement: Multimedia Appendix 2 [file formative_v7i1e41388_app2.docx]

| **Article Title** | **Journal** | **Altmetric Attention Score** | **Citation Count** | **Article Type** | **# News Mentions** | **# Blog Mentions** | **# Policy Mentions** | **# Twitter**  **Mentions** | **# Facebook Mentions** | **# Wikipedia Mentions** | **# Reddit Mentions** | **# Mendeley Citations** | **# Dimensions**  **Citations** |
| --- | --- | --- | --- | --- | --- | --- | --- | --- | --- | --- | --- | --- | --- |
| The proximal origin of SARS-CoV-2 | Nature Medicine | 33828 | 30 | correspondence | 1055 | 92 | 0 | 84022 | 51 | 8 | 25 | 0 | 108 |
| Aerosol and Surface Stability of SARS-CoV-2 as Compared with SARS-CoV-1 | New England Journal of Medicine | 23667 | 141 | correspondence | 2021 | 131 | 6 | 25776 | 34 | 3 | 25 | 0 | 289 |
| Substantial undocumented infection facilitates the rapid dissemination of novel coronavirus (SARS-CoV-2) | Science | 17114 | 54 | original | 632 | 68 | 4 | 24778 | 16 | 3 | 24 | 0 | 174 |
| Covid-19 - Navigating the Uncharted | New England Journal of Medicine | 15962 | 35 | editorial | 485 | 35 | 2 | 28187 | 38 | 2 | 27 | 0 | 84 |
| Treatment of 5 Critically Ill Patients With COVID-19 With Convalescent Plasma | JAMA: Journal of the American Medical Association | 14971 | 24 | original | 244 | 23 | 0 | 43895 | 6 | 1 | 18 | 796 | 70 |
| Clinical features of patients infected with 2019 novel coronavirus in Wuhan, China | The Lancet | 14276 | 1096 | original | 1169 | 101 | 21 | 19549 | 49 | 11 | 20 | 0 | 2233 |
| Clinical course and risk factors for mortality of adult inpatients with COVID-19 in Wuhan, China: a retrospective cohort study | The Lancet | 12956 | 252 | original | 927 | 56 | 2 | 15625 | 16 | 0 | 14 | 0 | 654 |
| COVID-19 Outbreak Associated with Air Conditioning in Restaurant, Guangzhou, China, 2020 | Emerging Infectious Diseases | 12749 | 1 | correspondence | 301 | 9 | 0 | 26854 | 3 | 1 | 14 | 0 | 2 |
| Persistence of coronaviruses on inanimate surfaces and their inactivation with biocidal agents | Journal of Hospital Infection | 12520 | 80 | systematic review | 743 | 28 | 8 | 14401 | 40 | 4 | 40 | 0 | 140 |
| The FDA-approved drug ivermectin inhibits the replication of SARS-CoV-2 in vitro | Antiviral Research | 12244 | 7 | original | 300 | 14 | 0 | 18697 | 9 | 3 | 29 | 0 | 23 |
| Characteristics of and Important Lessons From the Coronavirus Disease 2019 (COVID-19) Outbreak in China | JAMA: Journal of the American Medical Association | 11631 | 334 | viewpoint | 1079 | 97 | 7 | 18184 | 49 | 1 | 11 | 2581 | 737 |
| Severe Outcomes Among Patients with Coronavirus Disease 2019 (COVID-19) ó United States, February 12-March 16, 2020 | MMWR: Morbidity & Mortality Weekly Report | 11103 | 25 | correspondence | 1164 | 78 | 13 | 11866 | 11 | 0 | 14 | 0 | 70 |
| Temporal dynamics in viral shedding and transmissibility of COVID-19 | Nature Medicine | 10357 | 4 | correspondence | 324 | 13 | 0 | 15427 | 1 | 2 | 7 | 0 | 25 |
| Clinical Characteristics of Coronavirus Disease 2019 in China | New England Journal of Medicine | 10260 | 521 | original | 635 | 79 | 21 | 26495 | 35 | 2 | 11 | 0 | 1121 |
| Transmission of 2019-nCoV Infection from an Asymptomatic Contact in Germany | New England Journal of Medicine | 9421 | 214 | correspondence | 346 | 44 | 4 | 12645 | 33 | 2 | 15 | 0 | 427 |
| Offline: COVID-19 and the NHS-“a national scandal” | The Lancet | 8781 | 3 | editorial | 140 | 6 | 0 | 17315 | 0 | 1 | 5 | 0 | 4 |
| Compassionate Use of Remdesivir for Patients with Severe Covid-19 | New England Journal of Medicine | 8686 | 5 | original | 434 | 26 | 0 | 11366 | 8 | 1 | 11 | 0 | 39 |
| The Incubation Period of Coronavirus Disease 2019 (COVID-19) From Publicly Reported Confirmed Cases: Estimation and Application | Annals of Internal Medicine | 8544 | 64 | original | 1176 | 60 | 2 | 5114 | 12 | 0 | 0 | 0 | 186 |
| Do us a favor | Science | 8253 | 3 | editorial | 48 | 5 | 0 | 18572 | 14 | 0 | 15 | 0 | 1 |
| High Contagiousness and Rapid Spread of Severe Acute Respiratory Syndrome Coronavirus 2 | Emerging Infectious Diseases | 8211 | 5 | original | 114 | 8 | 0 | 14274 | 4 | 3 | 30 | 0 | 17 |
| A Trial of Lopinavir-Ritonavir in Adults Hospitalized with Severe Covid-19 | New England Journal of Medicine | 8059 | 102 | original | 385 | 49 | 1 | 9261 | 24 | 6 | 8 | 0 | 246 |
| Response to COVID-19 in Taiwan | JAMA: Journal of the American Medical Association | 7964 | 27 | viewpoint | 389 | 22 | 1 | 11106 | 7 | 1 | 21 | 466 | 67 |
| Hydroxychloroquine and azithromycin as a treatment of COVID-19: results of an open-label non-randomized clinical trial | International Journal of Antimicrobial Agents | 7869 | 60 | original | 417 | 58 | 0 | 9222 | 9 | 3 | 3 | 0 | 244 |
| Turbulent Gas Clouds and Respiratory Pathogen Emissions | JAMA: Journal of the American Medical Association | 7171 | 10 | editorial | 498 | 32 | 0 | 7427 | 3 | 5 | 13 | 269 | 26 |
| Remdesivir and chloroquine effectively inhibit the recently emerged novel coronavirus (2019-nCoV) in vitro | Cell Research | 6979 | 201 | correspondence | 393 | 59 | 1 | 9421 | 23 | 8 | 14 | 0 | 425 |
| Are patients with hypertension and diabetes mellitus at increased risk for COVID-19 infection? | The Lancet Respiratory Medicine | 6962 | 71 | correspondence | 328 | 34 | 0 | 7125 | 19 | 2 | 23 | 0 | 155 |
| Phylogenetic network analysis of SARS-CoV-2 genomes | Proceedings of the National Academy of Sciences of the United States of America | 6817 | 1 | correspondence | 318 | 12 | 0 | 9306 | 3 | 2 | 15 | 0 | 13 |
| Public Health Responses to COVID-19 Outbreaks on Cruise Ships Worldwide, February-March 2020 | MMWR: Morbidity & Mortality Weekly Report | 6796 | 3 | correspondence | 562 | 29 | 1 | 7543 | 6 | 3 | 4 | 0 | 13 |
| Epidemiology of COVID-19 Among Children in China | Pediatrics | 6667 | 52 | original | 566 | 35 | 1 | 6429 | 6 | 0 | 5 | 0 | 113 |
| Early Transmission Dynamics in Wuhan, China, of Novel Coronavirus Infected Pneumonia | New England Journal of Medicine | 6616 | 556 | original | 428 | 59 | 13 | 6910 | 29 | 9 | 6 | 0 | 1187 |
| A Trial of Lopinavir–Ritonavir in Adults Hospitalized with Severe Covid-19 | New England Journal of Medicine | 6444 | 102 | original | 154 | 15 | 0 | 8033 | 11 | 6 | 7 | 0 | 1 |
| Presenting Characteristics, Comorbidities, and Outcomes Among 5700 Patients Hospitalized With COVID-19 in the New York City Area | JAMA: Journal of the American Medical Association | 6289 | 2 | original | 322 | 25 | 0 | 8361 | 2 | 0 | 9 | 278 | 9 |
| Responding to Covid-19 - A Once-in-a-Century Pandemic? | New England Journal of Medicine | 6010 | 18 | viewpoint | 323 | 28 | 0 | 6137 | 19 | 2 | 21 | 0 | 38 |
| Virological assessment of hospitalized patients with COVID-2019 | Nature | 5958 | -99 | correspondence | 225 | 20 | 0 | 10275 | 2 | 2 | 8 | 0 | 84 |
| Coronavirus Disease Outbreak in Call Center, South Korea | Emerging Infectious Diseases | 5941 | -99 | original | 63 | 3 | 0 | 11045 | 0 | 0 | 4 | 0 | 0 |
| COVID-19 and Italy: what next? | The Lancet | 5872 | 71 | viewpoint | 192 | 20 | 1 | 7037 | 11 | 1 | 8 | 0 | 119 |
| Ten Weeks to Crush the Curve | New England Journal of Medicine | 5814 | -99 | editorial | 106 | 5 | 0 | 8445 | 3 | 0 | 3 | 0 | 2 |
| Early Transmission Dynamics in Wuhan, China, of Novel Coronavirus–Infected Pneumonia | New England Journal of Medicine | 5762 | 556 | original | 309 | 39 | 1 | 6296 | 27 | 6 | 5 | 165 | 327 |
| Clinical Characteristics of 138 Hospitalized Patients With 2019 Novel Coronavirus–Infected Pneumonia in Wuhan, China | JAMA: Journal of the American Medical Association | 5759 | 647 | original | 370 | 25 | 1 | 6725 | 23 | 1 | 10 | 125 | 222 |
| COVID-19 outbreak on the Diamond Princess cruise ship: estimating the epidemic potential and effectiveness of public health countermeasures | Journal of Travel Medicine | 5684 | 11 | original | 29 | 7 | 0 | 29161 | 2 | 1 | 4 | 0 | 28 |
| Covid-19: four fifths of cases are asymptomatic, China figures indicate | British Medical Journal | 5580 | 4 | editorial | 90 | 13 | 0 | 7938 | 6 | 1 | 11 | 0 | 8 |
| Cross species transmission of the newly identified coronavirus 2019-nCoV | Journal of Medical Virology | 5573 | 66 | original | 456 | 34 | 1 | 6451 | 19 | 0 | 2 | 0 | 120 |
| No evidence of rapid antiviral clearance or clinical benefit with the combination of hydroxychloroquine and azithromycin in patients with severe COVID-19 infection | Medecine & Maladies Infectieuses | 5557 | 12 | correspondence | 120 | 22 | 0 | 8674 | 2 | 0 | 13 | 0 | 29 |
| The psychological impact of quarantine and how to reduce it: rapid review of the evidence | The Lancet | 5383 | 64 | review | 422 | 45 | 0 | 4537 | 12 | 4 | 15 | 0 | 137 |
| How will country-based mitigation measures influence the course of the COVID-19 epidemic? | The Lancet | 5364 | 38 | editorial | 138 | 29 | 2 | 6611 | 9 | 8 | 4 | 0 | 103 |
| SARS-CoV-2 Infection in Children | New England Journal of Medicine | 5292 | 32 | correspondence | 134 | 10 | 1 | 10107 | 8 | 0 | 4 | 0 | 74 |
| Spread of SARS-CoV-2 in the Icelandic Population | New England Journal of Medicine | 5211 | 0 | original | 367 | 14 | 0 | 5492 | 3 | 1 | 3 | 0 | 17 |
| SARS-CoV-2 Viral Load in Upper Respiratory Specimens of Infected Patients | New England Journal of Medicine | 5086 | 143 | correspondence | 265 | 15 | 1 | 10664 | 8 | 1 | 1 | 0 | 345 |
| Nowcasting and forecasting the potential domestic and international spread of the 2019-nCoV outbreak originating in Wuhan, China: a modelling study | The Lancet | 4980 | 170 | original | 429 | 26 | 0 | 4704 | 9 | 4 | 20 | 56 | 109 |
| Estimates of the severity of coronavirus disease 2019: a model-based analysis | Lancet Infectious Diseases | 4947 | 14 | original | 375 | 41 | 2 | 4855 | 3 | 1 | 8 | 0 | 77 |
| Fair Allocation of Scarce Medical Resources in the Time of Covid-19 | New England Journal of Medicine | 4898 | 38 | editorial | 195 | 43 | 0 | 7620 | 0 | 0 | 6 | 0 | 94 |
| Effectiveness of convalescent plasma therapy in severe COVID-19 patients | Proceedings of the National Academy of Sciences of the United States of America | 4894 | 7 | original | 202 | 14 | 0 | 7463 | 1 | 1 | 3 | 0 | 21 |
| A Novel Coronavirus from Patients with Pneumonia in China, 2019 | New England Journal of Medicine | 4805 | 664 | original | 171 | 37 | 2 | 7516 | 50 | 6 | 1 | 0 | 1447 |
| Statement in support of the scientists, public health professionals, and medical professionals of China combatting COVID-19 | The Lancet | 4667 | 8 | correspondence | 280 | 15 | 0 | 6233 | 2 | 0 | 7 | 0 | 10 |
| Viral dynamics in mild and severe cases of COVID-19 | Lancet Infectious Diseases | 4585 | 18 | correspondence | 109 | 9 | 1 | 9461 | 0 | 0 | 6 | 0 | 42 |
| A pneumonia outbreak associated with a new coronavirus of probable bat origin | Nature | 4580 | 401 | original | 591 | 47 | 0 | 2725 | 20 | 10 | 5 | 0 | 875 |
| Aerosol and Surface Distribution of Severe Acute Respiratory Syndrome Coronavirus 2 in Hospital Wards, Wuhan, China, 2020 | Emerging Infectious Diseases | 4537 | 2 | original | 242 | 10 | 0 | 4856 | 4 | 0 | 8 | 0 | 11 |
| Epidemiological and clinical characteristics of 99 cases of 2019 novel coronavirus pneumonia in Wuhan, China: a descriptive study | The Lancet | 4537 | 596 | original | 372 | 33 | 10 | 3987 | 9 | 5 | 10 | 0 | 1197 |
| A familial cluster of pneumonia associated with the 2019 novel coronavirus indicating person-to-person transmission: a study of a family cluster | The Lancet | 4529 | 423 | original | 282 | 25 | 14 | 4935 | 8 | 4 | 4 | 0 | 736 |
| School closure and management practices during coronavirus outbreaks including COVID-19: a rapid systematic review | The Lancet Child & Adolescent Health | 4426 | 0 | review | 238 | 10 | 0 | 4923 | 1 | 0 | 1 | 0 | 3 |
| Presumed Asymptomatic Carrier Transmission of COVID-19 | JAMA: Journal of the American Medical Association | 4405 | 109 | correspondence | 286 | 26 | 0 | 4747 | 12 | 0 | 5 | 1174 | 262 |
| Epidemiology and transmission of COVID-19 in 391 cases and 1286 of their close contacts in Shenzhen, China: a retrospective cohort study | Lancet Infectious Diseases | 4396 | 1 | original | 128 | 3 | 0 | 6386 | 2 | 0 | 1 | 0 | 0 |
| Breakthrough: Chloroquine phosphate has shown apparent efficacy in treatment of COVID-19 associated pneumonia in clinical studies | BioScience Trends | 4335 | 112 | editorial | 155 | 16 | 1 | 5385 | 17 | 1 | 3 | 0 | 210 |
| Presymptomatic Transmission of SARS-CoV-2 in Singapore, January 23-March 16, 2020 | MMWR: Morbidity & Mortality Weekly Report | 4297 | 9 | correspondence | 422 | 25 | 0 | 3723 | 7 | 0 | 4 | 0 | 37 |
| The Effect of Chloroquine, Hydroxychloroquine and Azithromycin on the Corrected QT Interval in Patients with SARS-CoV-2 Infection | Circulation: Arrhythmia and Electrophysiology | 4229 | 0 | original | 1 | 1 | 0 | 22468 | 0 | 0 | 5 | 0 | 0 |
| Genomic characterisation and epidemiology of 2019 novel coronavirus: implications for virus origins and receptor binding | The Lancet | 4073 | 353 | original | 228 | 24 | 0 | 5547 | 15 | 5 | 2 | 0 | 668 |
| Pharmacologic Treatments for Coronavirus Disease 2019 (COVID-19) | JAMA: Journal of the American Medical Association | 4036 | 5 | original | 30 | 1 | 0 | 7074 | 11 | 2 | 1 | 753 | 10 |
| Microneedle array delivered recombinant coronavirus vaccines: Immunogenicity and rapid translational development | EBioMedicine | 4018 | 1 | original | 372 | 19 | 0 | 2506 | 2 | 1 | 4 | 0 | 7 |
| Clinical characteristics and intrauterine vertical transmission potential of COVID-19 infection in nine pregnant women: a retrospective review of medical records | The Lancet | 3989 | 138 | original | 382 | 40 | 3 | 9767 | 58 | 1 | 3 | 0 | 273 |
| Covid-19: ibuprofen should not be used for managing symptoms, say doctors and scientists | British Medical Journal | 3971 | 12 | editorial | 80 | 12 | 1 | 5793 | 21 | 0 | 9 | 0 | 24 |
| SARS-CoV-2 Cell Entry Depends on ACE2 and TMPRSS2 and Is Blocked by a Clinically Proven Protease Inhibitor | Cell | 3964 | 113 | original | 156 | 27 | 0 | 5813 | 15 | 3 | 33 | 0 | 377 |
| Preliminary Estimates of the Prevalence of Selected Underlying Health Conditions Among Patients with Coronavirus Disease 2019 in United States, February 12-March 28, 2020 | MMWR: Morbidity & Mortality Weekly Report | 3951 | 7 | correspondence | 358 | 23 | 0 | 3275 | 4 | 0 | 5 | 0 | 38 |
| Experimental Treatment with Favipiravir for COVID-19: An Open-Label Control Study | Engineering | 3777 | 6 | original | 11 | 0 | 0 | 10115 | 0 | 1 | 2 | 0 | 14 |
| Rational use of face masks in the COVID-19 pandemic | The Lancet Respiratory Medicine | 3704 | 11 | editorial | 180 | 19 | 0 | 5366 | 3 | 1 | 8 | 0 | 25 |
| Estimating the asymptomatic proportion of coronavirus disease 2019 (COVID-19) cases on board the Diamond Princess cruise ship, Yokohama, Japan, 2020 | Eurosurveillance | 3638 | 26 | original | 476 | 27 | 1 | 1381 | 2 | 0 | 16 | 0 | 102 |
| Susceptibility of ferrets, cats, dogs, and other domesticated animals to SARS-coronavirus 2 | Science | 3625 | 1 | original | 247 | 41 | 0 | 2545 | 4 | 4 | 5 | 0 | 16 |
| Case-Fatality Rate and Characteristics of Patients Dying in Relation to COVID-19 in Italy | JAMA: Journal of the American Medical Association | 3566 | 51 | viewpoint | 39 | 11 | 0 | 6379 | 4 | 0 | 2 | 804 | 140 |
| The effect of travel restrictions on the spread of the 2019 novel coronavirus (COVID-19) outbreak | Science | 3540 | 19 | original | 224 | 25 | 0 | 3539 | 8 | 5 | 9 | 0 | 91 |
| The neuroinvasive potential of SARS-CoV2 may play a role in the respiratory failure of COVID?19 patients | Journal of Medical Virology | 3423 | 43 | review | 118 | 8 | 0 | 4499 | 7 | 0 | 36 | 0 | 89 |
| Update: Public Health Response to the Coronavirus Disease 2019 Outbreak in United States, February 24, 2020 | MMWR: Morbidity & Mortality Weekly Report | 3393 | 6 | correspondence | 273 | 13 | 1 | 3410 | 13 | 0 | 2 | 0 | 29 |
| Antibody responses to SARS-CoV-2 in patients with COVID-19 | Nature Medicine | 3324 | 0 | original | 17 | 0 | 0 | 8731 | 5 | 0 | 8 | 0 | 0 |
| Asymptomatic Transmission, the Achillesí Heel of Current Strategies to Control Covid-19 | New England Journal of Medicine | 3321 | 0 | editorial | 76 | 4 | 0 | 4542 | 1 | 0 | 10 | 0 | 1 |
| COVID-19: protecting health-care workers | The Lancet | 3300 | 18 | editorial | 94 | 6 | 0 | 6826 | 4 | 0 | 0 | 0 | 25 |
| Cryo-EM structure of the 2019-nCoV spike in the prefusion conformation | Science | 3294 | 89 | report | 156 | 21 | 0 | 3590 | 16 | 0 | 6 | 7 | 31 |
| Estimating clinical severity of COVID-19 from the transmission dynamics in Wuhan, China | Nature Medicine | 3291 | 12 | correspondence | 231 | 46 | 0 | 3829 | 1 | 1 | 7 | 0 | 51 |
| Hydroxychloroquine, a less toxic derivative of chloroquine, is effective in inhibiting SARS-CoV-2 infection in vitro | Cell Discovery | 3244 | 32 | correspondence | 100 | 2 | 0 | 3664 | 4 | 0 | 16 | 0 | 0 |
| Positive RT-PCR Test Results in Patients Recovered From COVID-19 | JAMA: Journal of the American Medical Association | 3241 | 33 | correspondence | 133 | 7 | 0 | 3691 | 13 | 0 | 5 | 653 | 66 |
| Critical Care Utilization for the COVID-19 Outbreak in Lombardy, Italy | JAMA: Journal of the American Medical Association | 3182 | 51 | viewpoint | 115 | 16 | 0 | 3925 | 5 | 0 | 1 | 392 | 115 |
| Large-Vessel Stroke as a Presenting Feature of Covid-19 in the Young | New England Journal of Medicine | 3152 | 0 | correspondence | 239 | 4 | 0 | 2343 | 2 | 0 | 6 | 0 | 0 |
| Update: Public Health Response to the Coronavirus Disease 2019 Outbreak — United States, February 24, 2020 | MMWR: Morbidity & Mortality Weekly Report | 3147 | 6 | correspondence | 225 | 13 | 1 | 3307 | 12 | 0 | 2 | 1 | 4 |
| Changes in contact patterns shape the dynamics of the COVID-19 outbreak in China | Science | 3107 | 0 | correspondence | 100 | 4 | 0 | 5396 | 1 | 0 | 4 | 0 | 0 |
| Novel Coronavirus Infection in Hospitalized Infants Under 1 Year of Age in China | JAMA: Journal of the American Medical Association | 3095 | 50 | correspondence | 169 | 8 | 0 | 13087 | 8 | 0 | 2 | 9 | 13 |
| Breadth of concomitant immune responses prior to patient recovery: a case report of non-severe COVID-19 | Nature Medicine | 3093 | 10 | correspondence | 171 | 12 | 0 | 4886 | 3 | 0 | 5 | 0 | 28 |
| Aerosol Filtration Efficiency of Common Fabrics Used in Respiratory Cloth Masks | ACS Nano | 2958 | 0 | original | 156 | 13 | 0 | 3553 | 1 | 0 | 5 | 0 | 0 |
| Cleaning and Disinfectant Chemical Exposures and Temporal Associations with COVID-19 ó National Poison Data System, United States, January 1, 2020-March 31, 2020 | MMWR: Morbidity & Mortality Weekly Report | 2956 | 0 | correspondence | 370 | 14 | 0 | 1524 | 8 | 0 | 4 | 0 | 0 |
| Endothelial cell infection and endotheliitis in COVID-19 | The Lancet | 2928 | 1 | correspondence | 71 | 4 | 0 | 10725 | 2 | 0 | 5 | 0 | 4 |
| CRISPRñCas12-based detection of SARS-CoV-2 | Nature Biotechnology | 2876 | 0 | original | 114 | 10 | 0 | 3846 | 7 | 0 | 13 | 0 | 2 |
| Factors Associated With Mental Health Outcomes Among Health Care Workers Exposed to Coronavirus Disease 2019 | JAMA Network Open | 2870 | 28 | original | 200 | 23 | 0 | 2204 | 4 | 0 | 3 | 754 | 64 |
| Coronavirus Disease 2019 in Children in United States, February 12-April 2, 2020 | MMWR: Morbidity & Mortality Weekly Report | 2844 | 0 | correspondence | 325 | 12 | 1 | 1962 | 3 | 0 | 3 | 0 | 9 |
| Use of Hydroxychloroquine and Chloroquine During the COVID-19 Pandemic: What Every Clinician Should Know | Annals of Internal Medicine | 2841 | 1 | editorial | 89 | 9 | 0 | 6021 | 1 | 0 | 0 | 0 | 8 |

Supplementary Table S2. Articles and Their Altmetric Score and Mentions
